# Consumer input into health care: Time for a new active and comprehensive model of consumer involvement

**DOI:** 10.1111/hex.12665

**Published:** 2018-03-07

**Authors:** Alix E. Hall, Jamie Bryant, Rob W. Sanson‐Fisher, Elizabeth A. Fradgley, Anthony M. Proietto, Ian Roos

**Affiliations:** ^1^ Health Behaviour Research Collaborative School of Medicine and Public Health Faculty of Health and Medicine University of Newcastle Callaghan NSW Australia; ^2^ Priority Research Centre for Health Behaviour University of Newcastle & Hunter Medical Research Institute University of Newcastle Callaghan NSW Australia; ^3^ Hunter Cancer Research Alliance Waratah NSW Australia; ^4^ Cancer Services and Cancer Network, Hunter New England Local Health District Newcastle NSW Australia; ^5^ Youth Research Centre Melbourne Graduate School of Education University of Melbourne Parkville Vic. Australia

**Keywords:** consumer involvement, consumer participation, health care quality, quality improvement

## Abstract

**Background:**

To ensure the provision of patient‐centred health care, it is essential that consumers are actively involved in the process of determining and implementing health‐care quality improvements. However, common strategies used to involve consumers in quality improvements, such as consumer membership on committees and collection of patient feedback via surveys, are ineffective and have a number of limitations, including: limited representativeness; tokenism; a lack of reliable and valid patient feedback data; infrequent assessment of patient feedback; delays in acquiring feedback; and how collected feedback is used to drive health‐care improvements.

**Objectives:**

We propose a new active model of consumer engagement that aims to overcome these limitations. This model involves the following: (i) the development of a new measure of consumer perceptions; (ii) low cost and frequent electronic data collection of patient views of quality improvements; (iii) efficient feedback to the health‐care decision makers; and (iv) active involvement of consumers that fosters power to influence health system changes.

## 1 INVOLVING CONSUMERS IN IMPROVING THE DELIVERY OF HEALTH CARE IS IMPORTANT

Incorporating consumer opinions and perspectives to inform the development and improvement of health‐care practice is becoming increasingly recognized as essential to the delivery of quality health care.^1^ The importance of consumer involvement in shaping health care is widely acknowledged and supported.^2^, ^3^ Across a number of countries, consumer involvement in determining and implementing quality improvements to health care is recommended.^4^, ^5^, ^6^ Likewise, in many countries, measures of health‐care quality now include patient experience as a key quality indicator.^4^, ^7^ Although it is recommended that consumer views be incorporated when making quality improvements to health care, there is little guidance about how to do this effectively.^1^, ^8^ Consequently, the method and degree to which consumers have been involved in improving quality of health care have varied widely.^6^, ^9^

## 2 CURRENT STRATEGIES FOR INVOLVING CONSUMERS IN QUALITY IMPROVEMENTS TO HEALTH CARE

There are a number of strategies that have been used to encourage consumer involvement in influencing health‐care quality improvement. The features, strengths and limitations of two common models are discussed in detail below.

### 2.1 Consumer representation on advisory councils, boards and committees

One of the primary ways consumers have been involved in health‐care quality improvements is by acting as representatives on advisory councils, committees and boards charged with making decisions about health‐care programmes, services or policies.^10^ Through participation on such committees, consumer representatives are often tasked with providing their views about particular issues with the aim of advocating for changes that will benefit the needs and interests of the wider population of consumers. The benefit of involving consumer representatives on advisory boards and committees is that they bring with them the unique first‐hand knowledge acquired through their experience of being an active user of the health‐care system. This unique perspective can extend the knowledge of health‐care providers and policymakers. This results in the consideration of wider and more diverse views and provides important insights that might otherwise be overlooked.^11^

However, involving only one or two consumer representatives on a board or committee may not always accurately represent the views of an entire cohort of consumers. Individual consumers have their own personal experiences of care which can be highly variable, and one consumer's personal views about quality improvement may conflict with those of others. In addition, a consumer will often represent specific demographic groups, which may limit the range of interests and diversity of experiences being represented for consideration in the decision‐making process.^12^ Certain segments of consumers can therefore be under‐represented when this model of consumer engagement is used to influence quality improvements. Men, those with rare forms of cancer, those who are socially disadvantaged and minority groups such as residents of rural communities and culturally and linguistically diverse populations are not well represented in these roles.^13^ Furthermore, involvement of consumers in this model often requires extensive time and commitment from the consumer representatives, which can be burdensome. This may again impact on the representativeness of the consumers involved, as only consumers who are highly motivated and have available time to contribute are likely to volunteer.

A second limitation of this approach is that consumer representative roles are often tokenistic, and their views can often be unheard. For this model of consumer engagement to be effective, the opinions of the consumer representatives must be reasonably considered by the committee or board.^14^ The extent to which consumer views are considered is usually at the discretion of health‐care staff and administrators.^15^ Unfortunately, there are reports that consumer views are often overlooked, downplayed and have little perceived influence on the decision‐making process.^15^ One of the biggest barriers impacting on whether consumer views are considered by decision makers is the perceived legitimacy and level of representativeness of the consumer by the other committee members.^14^, ^15^, ^16^ If other decision makers do not perceive the consumer to be representative of the wider population of consumers, committee leaders will often disregard or overrule the consumer's views.^3^, ^14^ Consumers may also feel hesitant to share their views in the presence of health‐care staff, due to concerns about how their views may be perceived and received.^15^ Attempts have been made to overcome these shortfalls, by encouraging health‐care staff to liaise and collaborate with larger consumer or “user” groups.^6^ However, similar concerns have been raised regarding the representativeness of such groups and their ability to influence the decision‐making process.^6^, ^15^

### 2.2 Patient satisfaction or experience surveys

An alternative model of consumer involvement is the collection and reporting of patient feedback to the health‐care system, usually via patient surveys. This model is conducted on the premise that informing health‐care services of patient views will instigate improvement based on those consumer views.^17^ Numerous measures exist to assess patient satisfaction, experiences and perceptions of quality of care. The use of patient surveys overcomes some of the limitations of using consumer representatives on committees or boards, by providing an opportunity to seek the perspective of a large and potentially representative sample of consumers. This model may also be less burdensome, as consumers can complete surveys as they wait for clinic appointments, or at another time that is convenient for them. Patient surveys are used regularly in a number of health‐care settings to drive health‐care quality improvements.^4^, ^7^, ^18^ However, research suggests that this model of consumer involvement does not always lead to improvements, with mixed results regarding its effectiveness and impact.^18^, ^19^ Some of the main limitations inhibiting the successful use of consumer feedback to drive quality improvements in health care are discussed below.

Long delays between data collection, analysis and feedback of data to decision makers have been identified as a significant barrier to the use of patient satisfaction and experience surveys in involving consumers in quality improvements.^18^, ^19^ Surveys are often conducted annually or biannually, which means that efficient and timely feedback to health‐care services is limited.^19^ Consequently, decision makers may disregard this type of consumer feedback on the basis that the data is out of date and is no longer relevant.^19^ Furthermore, the infrequency and delays between data collection make it difficult to assess the impact any quality improvements are having.^19^ It has been suggested that regular repeat patient surveys that allow continuous and quick feedback of results to the health‐care system would be more useful for driving quality improvements than providing annual snapshots.^18^

There are also a number of limitations associated with the processes used to interpret the data obtained through patient surveys and decide on which improvements are required. For one, there are often difficulties with involving all of the necessary personnel required to make effective improvements.^19^ For changes to be successfully implemented they usually require input and support from multiple personnel. Health‐care staff have also voiced concerns over a lack of expertise in statistics and knowledge of effective interventions to be able to interpret patient survey data and make appropriate quality improvements.^18^, ^19^ To overcome these issues, it has been suggested that health‐care staff be provided with direction and a standardized framework to guide them on how to interpret and effectively use patient data to make quality improvements.^18^ Furthermore, this model seldom involves consumers in determining what aspects of care will be improved.^3^, ^20^, ^21^ Frequently, the findings of such surveys are presented to health‐care administrators and health‐care providers, who are left to interpret and make decisions about the data.^10^ Consequently, consumer perceptions can easily be ignored or dismissed,^10^ and the improvements implemented may not be truly reflective of consumer's views.

There has been criticism about the rigour and usefulness of patient survey data.^18^ For instance, a systematic review concluded that the reliability and validity of many patient satisfaction measures were questionable, and their content validity was uncertain.^22^ The face validity of available tools may also be limited, with most having been developed with little to no involvement from consumers.^17^, ^23^ Patient satisfaction measures have also been considered conceptually complex 17 and lacking specificity.^18^, ^19^, ^24^ For instance, most patient surveys only ask for patient views on broad aspects of care, they are seldom asked to specify exactly what it is that they would like improved.^18^, ^24^ Further, some patient surveys only relate to an entire hospital or health‐care system, rather than a specific department.^18^ This lack of specificity can make it difficult for health‐care providers and administrators to interpret and decide what changes would be most useful and where.^19^ Ideally, consumer surveys should be valid, reliable and adequately cover all possible areas of care that could be improved (i.e comprehensive). They should also contain specific detail on exactly what changes patients would find most useful in improving their care (i.e specific detail).^24^ Finally, patient surveys should report the results by smaller units, such as a specific department.^18^

## 3 TIME FOR A NEW APPROACH?

While it is clearly recognized that consumer involvement is critical to ensuring high quality, patient‐centred health care, most current models of consumer involvement are ineffective.^14^ There have, however, been attempts to combine the two common models of consumer involvement discussed above to overcome their limitations and harness their strengths.^25^, ^26^ Most notably are attempts in Australia to improve mental health services, such as the Mental Health Consumer Perceptions and Experiences of Services (MH‐CoPES) Framework in New South Wales 26 and the Mental Health Experience Co‐design (MH ECO) in Victoria.^25^ Both frameworks use a feedback‐action model to collect regular feedback from consumers of mental health services and employ active engagement of consumers and health‐care staff to use this feedback to inform quality improvements and service evaluation. While both the MH‐CoPES and MH ECO present innovative models of consumer engagement for health‐care improvements, they do have a number of limitations. An important limitation is the use of paper‐based patient experience surveys that result in missing data and require extensive resources for data entry and analysis 27, 28 that can ultimately impact on efficient and timely feedback of data to services and consumers.^27^ The use of Computer Assisted Telephone Interviews (CATIs) and face‐to‐face focus groups by the MH ECO framework 25 again involves extensive resources for data entry and analysis, as well as data collection. The use of CATIs and focus groups can be quite burdensome for patients, with completion time taking up to half an hour for CATIs and 2 hours for focus groups.^25^ Patient feedback focuses on current patient experiences rather than specifically on the aspects of care that patients want improved,^28^ which can make it difficult for staff and consumers to confidently interpret and decide where quality improvements are perceived specifically by consumers as being needed.

**Figure 1:**
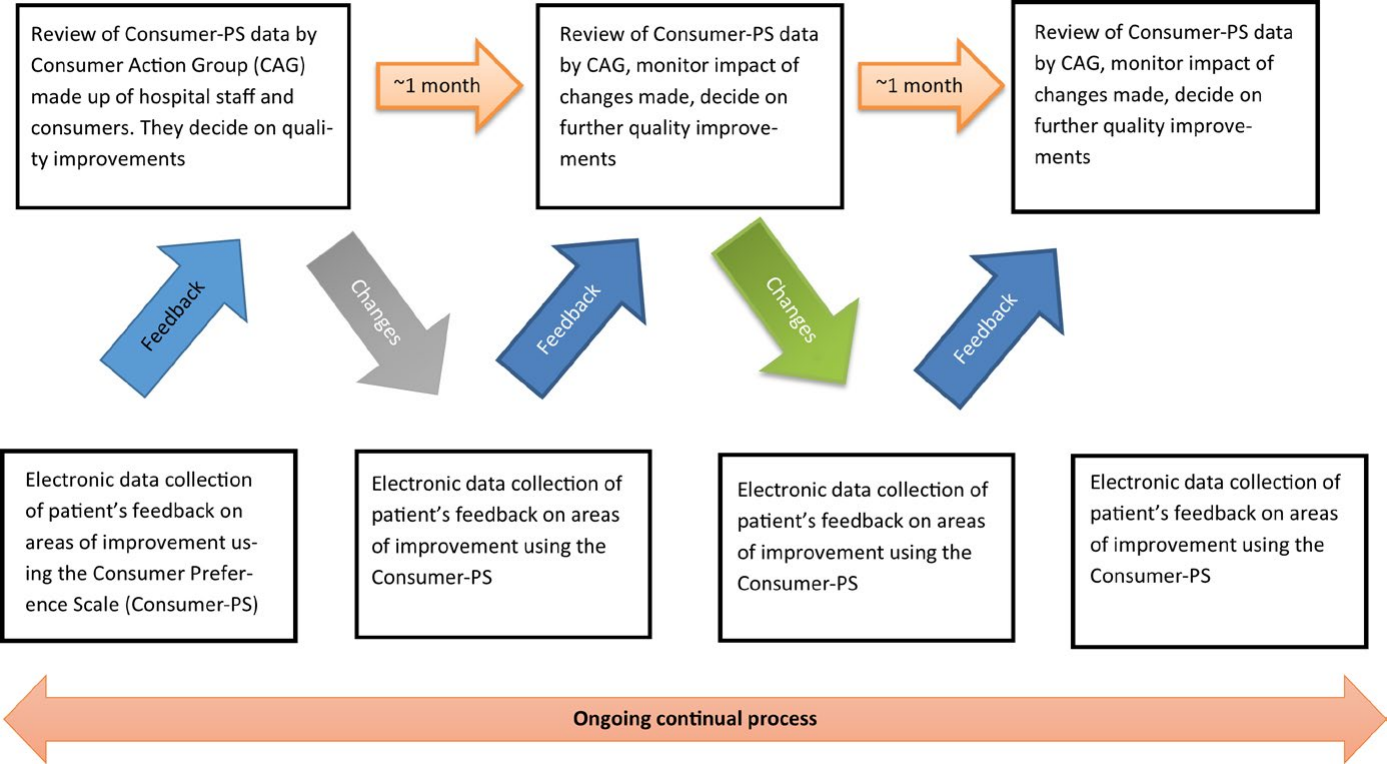
Overview of the Consumer Action Group model of consumer involvement to quality improvement to health care

We propose an innovative model of consumer involvement, which builds on the feedback and action models of MH‐CoPES and MH ECO, which we are evaluating (Trial registration number: ACTRN12614000702617). Like the MH‐CoPES and MH ECO models, our proposed model builds on the strengths of the current models of consumer engagement discussed above, while attempting to overcome their main limitations. However, with our model, we also strive to overcome the identified limitations of the MH‐CoPES and MH ECO frameworks, to design a model of consumer engagement model that can be efficiently employed by all types of health‐care services, not just mental health services. Our model consists of following two components (Figure 1):

1. Collection of data, from a representative sample of patients, at regular intervals, regarding the specific aspects of care they believe could be improved; and
2. Regular and ongoing feedback of this data, to a specially formed multidisciplinary Consumer Action Group (CAG), consisting of health‐care staff and consumer representatives, who will work together to develop and implement quality improvements to the issues identified by patients.

This model is underpinned by the following four mechanisms: (i) a comprehensive measure of consumer perceptions that captures desired quality improvements; (ii) low cost and frequent electronic data collection; (iii) an efficient theory‐driven method of feedback to the health‐care system; and (iv) active participation of consumers in instigating quality improvements.

### 3.1 A comprehensive measure of consumer perceptions

We have been working to overcome some of the main limitations inherent to patient feedback surveys by developing a new, comprehensive measure to elicit patient preferences for care across chronic disease groups and outpatient health service areas, known as the Consumer Preference Survey (Consumer‐PS).^24^ The Consumer‐PS is an online survey with interactive survey items. Consumers were extensively consulted and involved in the development of this measure.^24^ Participants are presented with a list of broad areas of health‐care services (e.g improved parking, information support, access to good hospital catering) and asked to indicate which areas they believe could be improved. Adaptive survey branching is then used to display more specific components of each of the areas of care patients previously identified as requiring improvement. For example, for the broad area of “improved parking,” more specific options such as “affordable options for parking,” “easy to use parking machines,” “reserve spaces for clinic patients only,” are displayed. Respondents then select which of these specific components of care they would find most useful if improved.^24^ Finally, patients are asked to complete a value‐weighting prioritization exercise where they select their top five broad areas for improvement. They are then asked to allocate a proportion of 100 points across their top five selected areas, to indicate which are most important to them, with a higher number of points indicating a higher level of importance. The Consumer‐PS has adequate face validity and test‐retest reliability for the majority of items. It takes <10 minutes to complete and has been found to be highly acceptable to patients and health‐care providers.^24^ While the Consumer‐PS still requires further refinement,^24^ unlike other patient feedback surveys, including that used by the MH‐CoPES framework which focused on patient experience,^28^ it provides a list of concise, specific and detailed targets for health service interventions.^24^ Further, the prioritization component indicates exactly what areas are most important to patients. The provision of such concrete and specific feedback from consumers eliminates the need for health‐care professionals to interpret and infer results.

### 3.2 Low cost and frequent electronic data collection

To ensure representative data are collected from consumers, low cost and efficient data collection methods, which cause minimal disruption to clinic functioning and little participant burden, are critical. Our model of consumer involvement utilizes an online platform to collect patient survey data using the Consumer‐PS. It is designed to be completed by all patients attending a specific hospital clinic or service (to ensure feedback from a representative sample is obtained), while they wait for their appointment or receive their treatment. Having a relatively brief survey that can be completed while patients attend their appointment will help to reduce consumer burden and reduce the low survey completion rates and high rates of missing data experienced by other models, such as the MH‐CoPES framework.^27^ Electronic data collection methods are also a simple and efficient method of data collection. They provide instantaneous access to the data collected in a usable form, as they do not require the data to be entered. This can reduce costs by eliminating the need for data entry personnel and participant follow‐ups associated with mailed or telephone surveys,^29^ such as those used by MH‐CoPES and MH ECO.^25^, ^26^, ^28^ Automated data entry can also reduce the time needed to prepare the data, which allows for more frequent and regular feedback of patient data to be provided to the health‐care system. This overcomes the current issues of providing infrequent, out‐of‐date patient data back to the hospital.^18^, ^19^ The sophisticated software programs used to support electronic data collection methods also allow the use of branching algorithms.^30^ The branching algorithms simplify the complexity of the Consumer‐PS by ensuring only relevant questions are provided to participants.^24^ This reduces participant burden as consumers are only required to answer questions that are relevant to them and has the potential to maximize data quality, such as less missing data. Finally, touch screen computer devices are appropriate for use in high volume, busy clinical settings and have been found to be highly acceptable by patients when compared to a pen‐and‐paper survey.^31^ By taking advantage of the many benefits of electronic data collection, our model of consumer involvement can routinely collect consumers’ views from a wide range of consumers across all demographics about the quality of health care and services, at a relatively low cost and with little burden on participants or the health‐care system. This is a significant advantage over similar feedback‐action models of consumer involvement to quality improvements, such as MH‐CoPES and MH ECO.

### 3.3 Efficient and regular feedback to the health‐care system

Passive, infrequent and inappropriate dissemination of data to health‐care professionals is unlikely to result in quality improvements. To ensure consumer feedback is optimally used by the health‐care system, it is critical that the following occur: (i) data are summarized in an easily understandable way; (ii) are fed back to the health‐care system in a continuous and frequent manner, preferably in real time as care is being delivered; (iii) are de‐identified to ensure individual patient answers are kept confidential, in order to help them feel that they can answer honestly; (iv) are provided to a range of health‐care professionals who will be responsible for instigating the relevant changes; and (v) the health‐care team have allocated time and dedicated support to review the data and make changes. In our proposed model of consumer involvement, a dedicated committee, known as the Consumer Action Group (CAG), is formed. This committee is founded on an adapted version of the Breakthrough Series model, which is a collaborative model of learning developed by the Institute for Healthcare Improvement.^32^ Within the model, a small dedicated team of professionals is formed to drive the quality improvement process. This team is responsible for setting goals and applying strategies for addressing identified gaps.^32^ They employ a collaborative and iterative process of implementing and evaluating their quality improvements, drawing on their expert knowledge and available evidence.^32^ They then implement their strategies, attempting small tests of change and measuring improvements.^32^

The Breakthrough Series model underpins the formation of the CAG. Specifically, the CAG consists of a small group of experts, which includes consumers (see below) and a variety of health‐care staff representing different levels of the hospital system. The different experts help to bring the necessary knowledge and personal experiences needed to develop quality improvement strategies based on best practice. Further, each member is chosen to represent the different levels of personnel required to implement quality improvements in a health‐care department. For example, a management level representative is involved as they are usually responsible for approving changes and allocating the necessary resources, while a range of clinical and support staff are included, as they are often responsible for implementing changes into practice. If possible, a staff member who is proactively supportive of the group should be included as a member. Ideally, the CAG meets regularly to examine the data collected from the Consumer‐PS, identify areas of need, set goals, and identify, implement and monitor improvement strategies to address the issues identified by patients. The use of the Breakthrough Series model provides the CAG members with a framework to guide them in their development and testing of strategies for quality improvement based on consumer feedback, something that has been missing or limited in previous models of consumer involvement.^18^, ^27^

Once a committee is formed, each hospital member is supported in their role by receiving an overview of the model and how to review and interpret the patient data. This provides an opportunity to create a shared knowledge and language within the CAG, which can help to reduce power imbalances and promote collegiality within the group.^33^ Offering support and training to hospital staff can increase their skills and confidence in interpreting patient data and using it to decide on appropriate quality improvements, an issue that has previously been identified as an area of concern when using patient feedback to guide quality improvements.^18^, ^19^ Training will also ensure that both consumers and hospital staff have the required skills to work together to review patient data, decide, implement and evaluate quality improvements. To further simplify the process and ensure the data are appropriately interpreted, a standardized template has been developed to present the Consumer‐PS data to this group. The template has been reviewed by consumers and health‐care providers. The use of a standardized template helps to solidify the shared language used by the group, by introducing an object that has shared meaning and knowledge attached to it.^33^

Once trained, the committee is encouraged to meet on a regular basis, preferably monthly. However, the exact timing of the meetings will depend on the needs of the health‐care department. The committee meetings are held face‐to‐face or via telephone, as a recent Cochrane review found that telephone and face‐to‐face group meetings engaged consumers better than mailed surveys when setting priorities for community health goals.^34^ During these meetings, the CAG is provided with an updated report on the Consumer‐PS data, which is collected from patients on an ongoing basis. This allows the group to identify areas for improvement that are relevant and timely, as well as monitor and evaluate whether the improvements made have had an impact on patient views.

### 3.4 Active participation of consumers

For consumers to truly be involved in making quality improvements to health care, they must be actively involved in deciding on what improvements are made and how. In our proposed model of consumer involvement, at least two dedicated consumer representatives are included as CAG members. The inclusion of more than one consumer increases the representativeness of the consumer voice, as well as providing the consumers with peer support amongst a team of health‐care staff.^16^ Consumers with experience of group committee processes have been found to be more influential in making decisions than those without such experience.^14^ Furthermore, consumers involved as a CAG representative will be required to commit a certain amount of their own time, on a regular basis, to help prepare for and attend CAG meetings. Consequently, this component of the model may induce a higher level of consumer burden, and thus, consumers included as CAG representatives must be willing and prepared for such a commitment. To increase the credibility of the consumer representatives, they should be selected through a competitive process, involving submission of an application, interview and training. This will ensure that the consumer representatives hold the necessary experience, qualities and commitment required for successful participation in the CAG. Once selected, the consumers are trained on the consumer model, including the principles of the Breakthrough Series model, the process involved in collecting patient responses to the Consumer‐PS, how to review and interpret the Consumer‐PS data using the standardized template, suggestions on how to decide on intervention strategies and how to monitor changes in the data. They also receive more generic training on communication skills, conflict resolution, decision‐making, the structure of the health‐care system and how to organize and chair meetings. Training also involves a component that is taught by a consumer representative involved in other health‐related committees and/or boards. This component is developed entirely by a consumer and is designed to provide first‐hand advice and skills on how to work as a consumer representative on a health‐related committee. The initial training is completed face‐to‐face with other consumer representatives. This allows the consumers to prepare for their role on the CAG, support relationships with other consumers, build confidence in their abilities 14 and develop a group language.^33^ This will further enhance the unity of the CAG as all members will be speaking a common and accepted language from the beginning.^33^ Important aspects of the training, such as conflict resolution and decision‐making, should be reiterated throughout consumer's time on the CAG.

To promote the active involvement of the consumers, they are allocated a key role within the CAG from the beginning. Such roles may include chair, time or minute keeper. To help to empower the consumer representatives and reduce any pre‐existing power imbalances that may be felt between the consumers and the health‐care staff, the chair of the CAG is always represented by one of the consumers. The legitimacy of the consumer's ability to represent the views of the wider population will be enhanced by the inclusion of the Consumer‐PS data, as the central focus of the CAG meetings. Using data collected from a wide, representative sample of patients to support consumer views has previously been found to add weight to consumer thoughts and to help stimulate discussions between consumer representatives and other committee members, particularly when a difference in opinion occurs.^14^ Finally, consumer representatives from the different hospital CAGs will meet semi‐regularly to share their experiences, knowledge and resources related to their efforts in making quality improvements. Consumers will be encouraged to share and apply the knowledge they gain from these meetings into the quality improvements they make within their own CAG. These “learning sessions” are based on the Breakthrough Series model 32 and differ from similar models of consumer involvement such as the MH‐CoPES model that does not include this process of sharing and learning between service, although they identify that such a process may be beneficial.^27^

## 4 CONCLUSION

The involvement of consumers is essential to ensuring patient‐centred health care is delivered. While there are a number of ways consumers can be involved in making health‐care quality improvements, the current models used are often ineffective and have multiple limitations. Furthermore, few studies have rigorously tested new models of consumer involvement. We have proposed a model of consumer involvement to health‐care quality improvement, which combines the strengths of previous models while addressing their limitations. Our model is based on the foundation of two core components: (i) the collection of accurate and representative views of consumers’ experiences of care; and (ii) the reporting of this data to a specially formed Consumer Action Group, which involves hospital staff and specially trained consumers working together to develop solutions to the issues identified by patients. Efforts are currently underway to implement and evaluate this new model.

## CONFLICT OF INTERESTS

The authors do not have any conflicts of interest to declare.
